# Direct phase selection of initial phases from single-wavelength anomalous dispersion (SAD) for the improvement of electron density and *ab initio* structure determination

**DOI:** 10.1107/S1399004714013868

**Published:** 2014-08-29

**Authors:** Chung-De Chen, Yen-Chieh Huang, Hsin-Lin Chiang, Yin-Cheng Hsieh, Hong-Hsiang Guan, Phimonphan Chuankhayan, Chun-Jung Chen

**Affiliations:** a Life Science Group, Scientific Research Division, National Synchrotron Radiation Research Center, 101 Hsin-Ann Road, Hsinchu 30076, Taiwan; b Department of Physics, National Tsing Hua University, Hsinchu, Taiwan; c Institute of Biotechnology, National Cheng Kung University, Tainan City 701, Taiwan; d The Center for Bioscience and Biotechnology, National Cheng Kung University, Tainan City 701, Taiwan

**Keywords:** direct phase selection, *ab initio* structure determination, electron-density improvement

## Abstract

Optimization of the initial phasing has been a decisive factor in the success of the subsequent electron-density modification, model building and structure determination of biological macromolecules using the single-wavelength anomalous dispersion (SAD) method. Two possible phase solutions (ϕ_1_ and ϕ_2_) generated from two symmetric phase triangles in the Harker construction for the SAD method cause the well known phase ambiguity. A novel direct phase-selection method utilizing the θ_DS_ list as a criterion to select optimized phases ϕ_am_ from ϕ_1_ or ϕ_2_ of a subset of reflections with a high percentage of correct phases to replace the corresponding initial SAD phases ϕ_SAD_ has been developed. Based on this work, reflections with an angle θ_DS_ in the range 35–145° are selected for an optimized improvement, where θ_DS_ is the angle between the initial phase ϕ_SAD_ and a preliminary density-modification (DM) phase ϕ_DM_^NHL^. The results show that utilizing the additional direct phase-selection step prior to simple solvent flattening without phase combination using existing DM programs, such as *RESOLVE* or *DM* from *CCP*4, significantly improves the final phases in terms of increased correlation coefficients of electron-density maps and diminished mean phase errors. With the improved phases and density maps from the direct phase-selection method, the completeness of residues of protein molecules built with main chains and side chains is enhanced for efficient structure determination.

## Abstract 2

A novel direct phase-selection method to select optimized phases from the ambiguous phases of a subset of reflections to replace the corresponding initial SAD phases has been developed. With the improved phases, the completeness of built residues of protein molecules is enhanced for efficient structure determination.

## 1. Introduction   

X-ray protein crystallography has been an efficient and dominant method for determining the three-dimensional structures of biological macromolecules. Despite great progress towards its automation, the phasing of diffraction reflections is still a key step for structure determination. The single-wavelength anomalous dispersion (SAD) method using S atoms and various heavy atoms in protein molecules has become increasingly important in phasing because protein crystals typically suffer from radiation damage during the collection of diffraction data by the commonly used multiple-wavelength anomalous dispersion (MAD) method. Moreover, S-MAD is not easily achievable at current synchrotron facilities because of its absorption edge in the low range of X-ray energies. The rate of success of S-SAD phasing is much more limited than the SAD method using heavy atoms (Hendrickson & Teeter, 1981 ▶; Dauter *et al.*, 1999 ▶; Liu *et al.*, 2000 ▶; Bond *et al.*, 2001 ▶; Cianci *et al.*, 2001 ▶; Gordon *et al.*, 2001 ▶).

The two main steps in structure determination using the SAD method with sulfur and heavy atoms are locating anomalous scattering atoms in the unit cell to obtain the initial SAD phases from the anomalous differences of structure factors from diffraction intensities and improving the phases and electron density from initial SAD phases by density modification with various algorithms. In general, the overall average figure of merit of the SAD phases is much smaller than that from MAD phasing. A powerful method of density modification or phase improvement following the initial SAD phasing is hence essential for the success of structure determination. Several density-modification approaches are available, such as solvent flattening (Wang, 1985 ▶), maximum entropy in the direct method (Bricogne, 1984 ▶, 1988 ▶), phase extension combined with entropy maximization and solvent flattening (Prince *et al.*, 1988 ▶), direct-space methods in phase extension and phase refinement (Refaat *et al.*, 1996 ▶), solvent flattening to improve the direct-method phases (Giacovazzo & Siliqi, 1997 ▶) and the programs *DM* from *CCP*4 (Cowtan & Main, 1993 ▶, 1996 ▶) and *RESOLVE* (Terwilliger, 2000 ▶).

For example, in the *SHELXC*/*D*/*E* program suite (Sheldrick, 2008 ▶), *SHELXC* is designed to provide a statistical analysis of the experimental X-ray diffraction data, to estimate the structure factors *F*_H_ of scattering atoms and to prepare the preliminary data for *SHELXD* and *SHELXE* to locate the positions of heavy atoms for initial phasing (Usón & Sheldrick, 1999 ▶; Sheldrick *et al.*, 2001 ▶; Schneider & Sheldrick, 2002 ▶) and to improve phases iteratively with density modification (Schneider & Sheldrick, 2002 ▶), respectively. The anomalous signals from heavy atoms can alternatively be refined iteratively with *Phaser* in *CCP*4 (McCoy *et al.*, 2007 ▶). The *CCP*4 program *DM* can further improve the initial experimental SAD phase to give an improved electron-density map (Cowtan & Main, 1993 ▶, 1996 ▶). The powerful software *SOLVE*/*RESOLVE* can accomplish all of the steps for macromolecular structure determination by the SAD method, including data scaling, location of heavy atoms, initial SAD phasing, density modification and model building. In SAD mode, initial phases are obtained with *SOLVE*; *RESOLVE* subsequently performs the identification of noncrystallographic symmetry (NCS; Terwilliger, 2002 ▶), density modification (Terwilliger, 2000 ▶) and automated model building (Terwilliger, 2003 ▶). After density modification with solvent flattening, solvent flipping, NCS averaging, histogram matching, maximum likelihood or entropy maximization, an additional step using *ARP*/*wARP* can substantially improve the phases (Perrakis *et al.*, 1997 ▶).

Beyond these protocols, some methods have been developed to resolve the phase ambiguity. One approach is to use the direct method based on the product of the Sim and Cochran distributions, which can improve the initial phases (Wang *et al.*, 2004 ▶). It has been shown that assigning accurate phases to a few strong reflections can improve the density-modification process in terms of the mean phase errors and map correlation coefficients (Vekhter, 2005 ▶). A recent study reported that the map skewness, which describes the extent to which the extreme values in a map tend to be systematically positive or negative, can be used to identify the correct phases for a few of the strongest reflections. A genetic algorithm was developed to optimize the quality of phases using the skewness of the density map as a target function. Such optimized phases have been used in density modification and the quality of the density maps was better than those generated from the original centroid phases (Uervirojnangkoorn *et al.*, 2013 ▶). The initial phases obtained from the SAD, SIRAS and SIR methods can be improved according to these two approaches.

In the present work, we focus mainly on the improvement of initial phases from the general SAD method using sulfur or heavy atoms based on a novel ‘direct phase-selection method’ based on a ‘θ_DS_ list’, where θ_DS_ is the angle between the initial SAD phase and the preliminary DM phase, differing from previously reported methods. We demonstrate that this method of phase selection can resolve the phase ambiguity and improve the phases from SAD with increased effectiveness in combination with *RESOLVE* or *DM* utilizing only simple solvent flattening without phase combination and an FOM cutoff. A number of experimental SAD data sets with sulfur or metal (Zn, Gd, Fe and Se) atoms as the anomalous scatterers in proteins have been tested, including two unknown new protein structures; all results show that superior phases can be obtained with this new phase-selection method, yielding an enhanced quality of the corresponding electron-density maps and increased completeness of model building.

## 2. The phase ambiguity of SAD   

**Figure 1:**
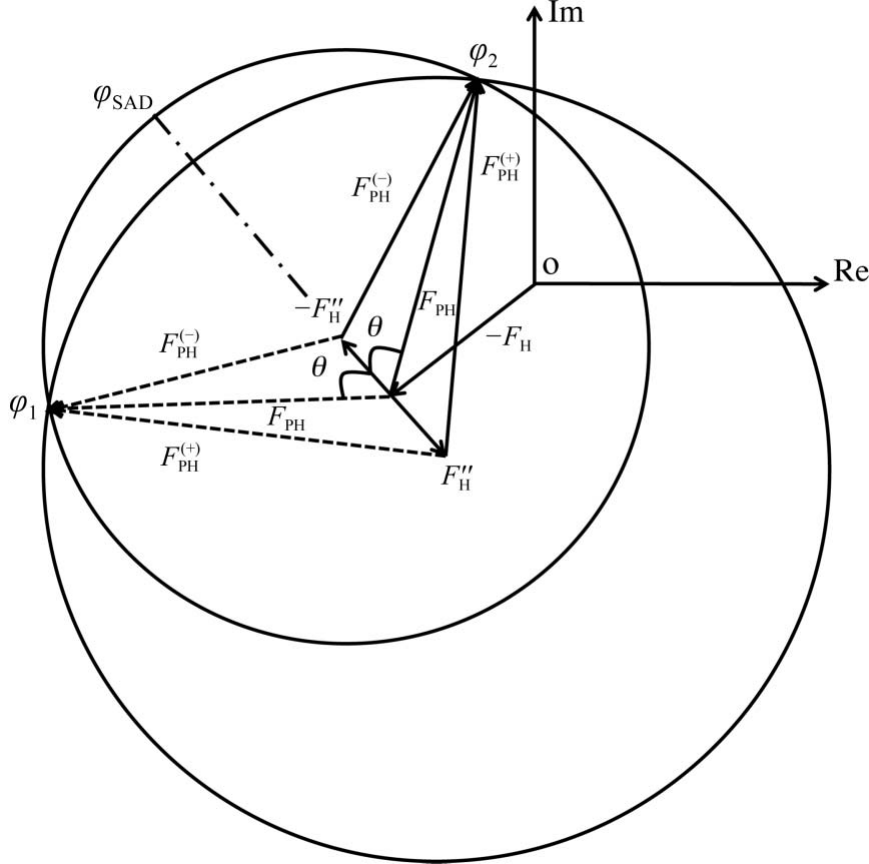
Harker construction for SAD phasing. The contribution of heavy atoms to a structure factor consists of a normal part, *F*_H_, and an anomalous part, *F*_H_′′. The structure factor *F*_PH_ is a normal part and *F*_PH_^+^ and *F*_PH_^−^ are anomalous parts of the protein crystal containing heavy atoms.

The SAD experiment provides measurements of anomalous signals or Bijvoet differences,
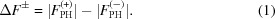
The amplitudes of the structure factors, 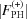 and 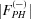, are measured from the diffraction intensities to estimate the contribution of anomalous scattering from heavy atoms. The positions of the heavy-atom substructures (*X*_H_) can be located with the direct method or the Patterson method to derive the heavy-atom substructure factors (*F*_H_) and the anomalous scattering contributions (*F*_H_′′). With this preliminary information, the Harker construction, which is based on the assumption that there are no errors in the amplitudes of structure factors or the heavy-atom model, generates two possible phase solutions (ϕ_1_ and ϕ_1_), with one being the true phase and the other a false phase, from two symmetric phase triangles, as shown in Fig. 1 ▶. This ‘phase ambiguity’ is a well known problem in protein crystallography, especially for the SAD method. The phase triangle shows that the structure factors *F*_H_′′ and *F*_PH_ are dependent on 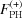 and 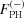, from which are derived
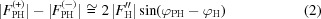
and
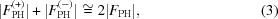
in which ϕ_PH_ and ϕ_H_ are the phases of *F*_PH_ and *F*_H_, respectively. |*F*_PH_| is used in the calculation of electron-density maps.

The phase ambiguity arises from the existence of an angle θ between *F*_PH_ and *F*_H_′′, related to 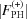, 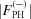 and |*F*_H_′′|, which can be calculated as (Blundell & Johnson, 1976 ▶)
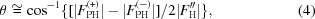
and
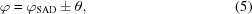
in which ϕ_SAD_ is the phase of *F*_H_′′.

## 3. Methods   

### 3.1. Crystal preparation and data collection   

Six SAD data sets were collected from five protein crystals with known structures, lysozyme_S (sulfur), lysozyme_Gd (gadolinium), insulin_S, lectin_Zn (zinc) and cytochrome *c*_3__Fe (iron), and one crystal of unknown structure, histidine-containing phosphotransfer B [HptB_Se (selenium)]. The crystallization of these proteins was performed by the hanging-drop vapour-diffusion method at 291 K. The crystallizations of lysozyme, insulin, lectin and cytochrome *c*_3_ were performed using previously described protocols (Nanao *et al.*, 2005 ▶; Nagem *et al.*, 2001 ▶; Huang *et al.*, 2006 ▶; Aragão *et al.*, 2003 ▶). Crystals of lysozyme_Gd and lectin_Zn were prepared with the soaking method, whereas HptB_Se was prepared with selenomethionine substitution during expression and crystallized (unpublished work). The X-ray SAD data sets were collected on beamline BL13B1 of the National Synchrotron Radiation Research Center (NSRRC) in Taiwan and beamline BL44XU of SPring-8 in Japan. The detailed statistics of data collection are summarized in Table 1 ▶.

### 3.2. Location of substructures and generation of initial SAD phases   

**Figure 2:**
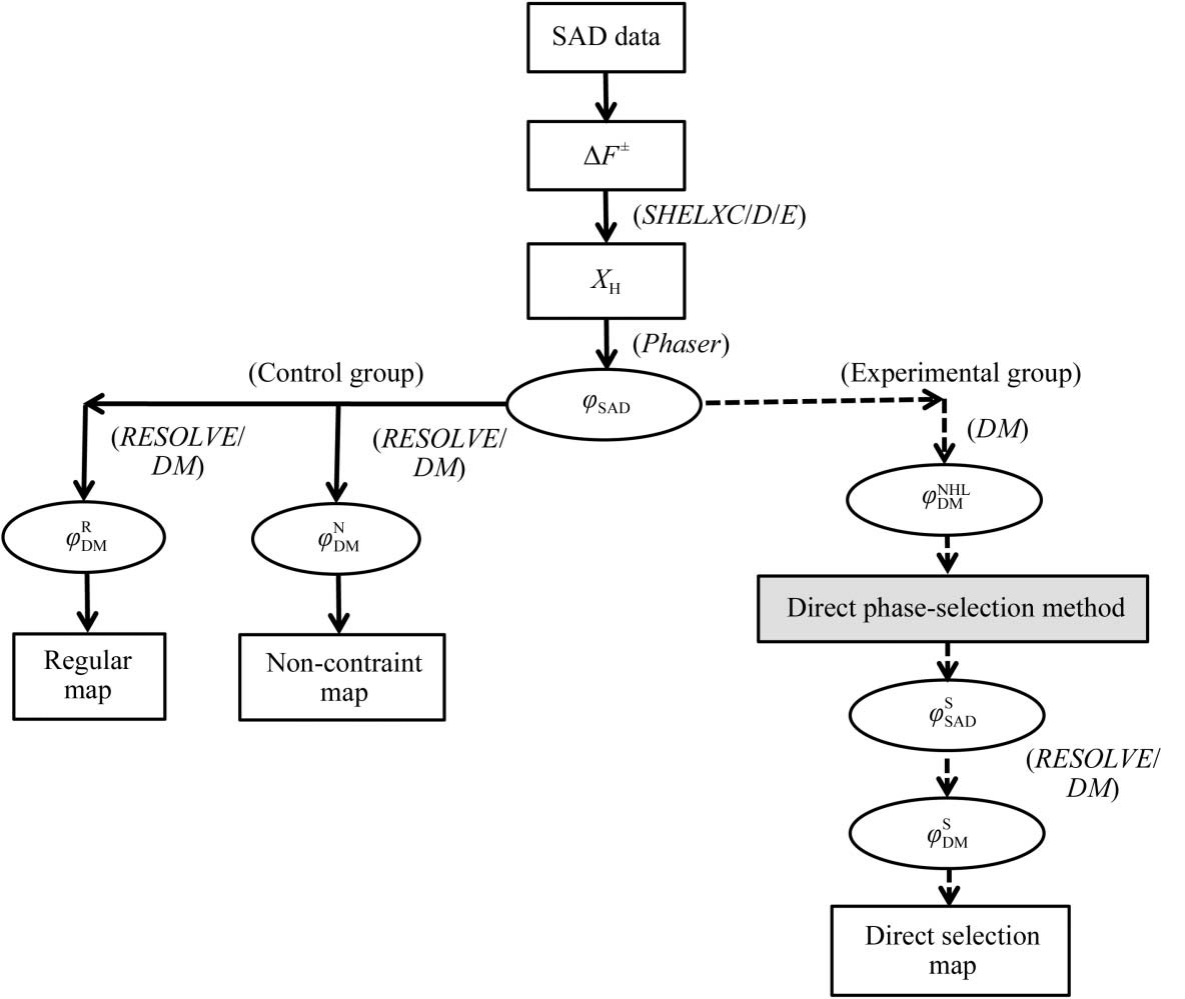
Flowchart of the direct phase-selection method combined with *RESOLVE* or *CCP*4. The corresponding programs are indicated in parentheses. The solid arrows show the commonly used phase methods and the dashed arrows show the new direct phase-selection method. The ellipses show the initial phases and DM phases for various methods; the rectangles show the anomalous SAD data (Δ*F* ^±^), the substructure positions of S and heavy atoms (*X*_H_) and various maps. The grey rectangle indicates the new direct phase-selection method.

**Figure 3:**
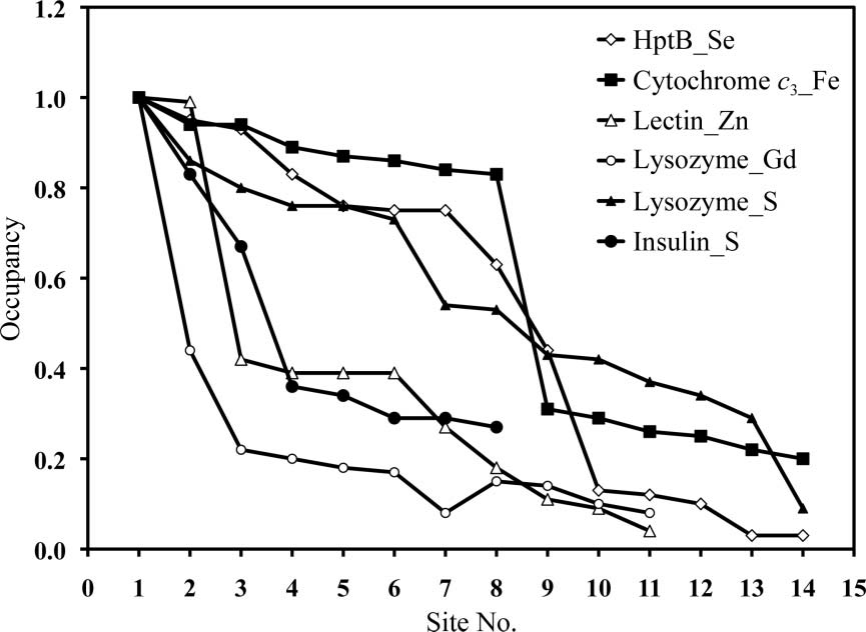
Locations of the heavy-atom sites with the corresponding occupancies calculated with *SHELXC*/*D*/*E* in *CCP*4.

The overall procedure of the new phase-selection method for phase improvement in this work is shown in Fig. 2 ▶. The details of the input and data for each program in all of the steps in this study are presented in Supplementary List S1.^1^ The S and heavy-atom substructures (*X*_H_) were determined from the anomalous SAD data (Δ*F* ^±^) with *SHELXC*/*D*/*E* in *CCP*4 (Sheldrick, 2008 ▶), which identified possible sites with high occupancies (Fig. 3 ▶). The positions and anomalous signals of S or heavy atoms were iteratively refined; the centroid phases were subsequently generated as the initial SAD phases (ϕ_SAD_) with *Phaser* in *CCP*4 (McCoy *et al.*, 2007 ▶).

### 3.3. The control group for commonly used procedures   

The flowchart of the overall procedure in this work is divisible into two groups: the control group (indicated by solid lines) and the experimental group (indicated by dashed lines) (Fig. 2 ▶). The control group consists of the regular method and the non-constraint method.

#### 3.3.1. Regular method   

In this step, we used *RESOLVE* to improve the initial SAD phase to obtain the final DM phases (ϕ^R^_DM_) and the regular map from the data set for the regular method, which is defined below, with the SAD standard protocol, including the Hendrickson–Lattman coefficients (phase probabilities) and fom_cut parameters, which set the initial resolution for density modification at the point at which the FOM has the default value of 0.15 (Terwilliger, 2000 ▶). For the parallel comparison, we also separately used the *CCP*4 program *DM* with solvent flattening and the standard parameters (Cowtan & Zhang, 1999 ▶), including the Hendrickson–Lattman coefficients with all reflections for the entire calculation (all reflections automatically weighted by the σ_A_ calculation were used in every cycle), for density modification from the same initial SAD phase. After these calculations, an adapted data set for the regular method was generated that included some important parameters from the mathematical operations, which include *hkl*, *F*_*hkl*_, ϕ_SAD_, initial FOM, ϕ^R^_DM_ (final DM phase from the regular method), θ, ϕ_1_ and ϕ_2_, and ϕ_C_ for further evaluation of the ‘percentage correct’. This process is called the ‘regular method’, and the corresponding electron-density map using the final DM phases ϕ^R^_DM_ is called the ‘regular map’.

For the theoretical simulation, the calculated model phases (ϕ_C_) were generated from the five corresponding refined structures. These initial structural models were obtained from the PDB (PDB entries 1gyo for cytochrome *c*_3_, 2bn3 for insulin and 2lyz for lysozyme) and our laboratory (lectin) and were refined with our experimental data with *REFMAC*5 (Murshudov *et al.*, 2011 ▶; Winn *et al.*, 2011 ▶) and visualized or adjusted with *Coot* (Emsley & Cowtan, 2004 ▶). The statistics of the structure refinement are summarized in Table 1 ▶.

#### 3.3.2. Non-constraint method   

For the non-constraint method, the final DM phases (ϕ^N^_DM_) were obtained using *RESOLVE* with the SAD standard default protocol, except for the Hendrickson–Lattman coefficients and fom_cut parameters, which set the initial resolution for density modification to the point at which the FOM value is 0. For a comparison, the *CCP*4 program *DM* was used in parallel to generate ϕ^N^_DM_ phases with standard default parameters and phase extension in FOM steps, in which only the low-resolution reflections were used in the first cycle and extra reflections were added in each cycle until all of the data were used. Histogram matching and the Hendrickson–Lattman coefficients were excluded. In other words, no phase combination was carried out, thus no Hendrickson–Lattman coefficients are provided; all of the data were used for density modification without a resolution cutoff in this method. After the above calculation with the non-constraint method, ϕ^N^_DM_ (the final DM phase from the non-constraint method) can be obtained. The phases ϕ^N^_DM_ and ϕ_C_ were later used for evalution of the ‘percentage correct’. This process is called the ‘non-constraint method’ and the corresponding electron-density map using final DM phases ϕ^N^_DM_ is called the ‘non-constraint map’. This calculation was performed for control purposes for comparison with the following experimental group with the same DM protocols.

### 3.4. The experimental group   

In the experimental group, the direct phase-selection method is utilized as a new algorithm to optimize the initial phases. In this new approach, density modification with simple solvent flattening is first used only once to select one of the two possible phase choices from the SAD phase probability distribution for a subset of the reflections where the phase choice is most likely to be correct, thus differing from the standard approaches using SAD phase combination with Hendrickson–Lattman coefficients throughout density modification.

#### 3.4.1. Preparation of data sets for the simulation test   

Among the six SAD experimental data sets, five cases, lysozyme_S, lysozyme_Gd, insulin_S, lectin_Zn and cytochrome *c*_3__Fe, were examined with calculated phases ϕ_C_ from their known models. The preliminary DM phases (ϕ^NHL^_DM_) were generated with one cycle of DM from the initial SAD phases ϕ_SAD_ using the *CCP*4 program *DM* with solvent flattening, histogram matching and all reflections for the entire calculation, involving no Hendrickson–Lattman coefficients (NHL). The important angle parameters were then generated in the data sets for examination by simulation, some of which differed from those in the data set for the regular method in §[Sec sec3.3]3.3, such as ϕ^NHL^_DM_, ϕ_am_ (ambiguity phase ϕ_1_ or ϕ_2_ determined from the preliminary DM phase ϕ^NHL^_DM_) and θ_DS_ (the angle between the initial SAD phase ϕ_SAD_ and the preliminary DM phase ϕ^NHL^_DM_).

#### 3.4.2. Overall procedures of the experimental group   

The experimental group in Fig. 2 ▶ shows the protocol to produce improved phases and direct selection maps. The optimum initial phases ϕ^S^_SAD_ were determined from ϕ^NHL^_DM_ by the direct phase-selection method *de novo* (see details in §§[Sec sec3.4.3]3.4.3 and [Sec sec3.4.4]3.4.4). The DM phase ϕ^S^_DM_ was subsequently improved from ϕ^S^_SAD_ using *RESOLVE* or *DM*, respectively, in parallel for comparison, using the same protocols as were used in the non-constraint method. The direct selection map is consequently generated with the final DM phases ϕ^S^_DM_.

#### 3.4.3. Phase-selection rule and definition   

**Figure 4:**
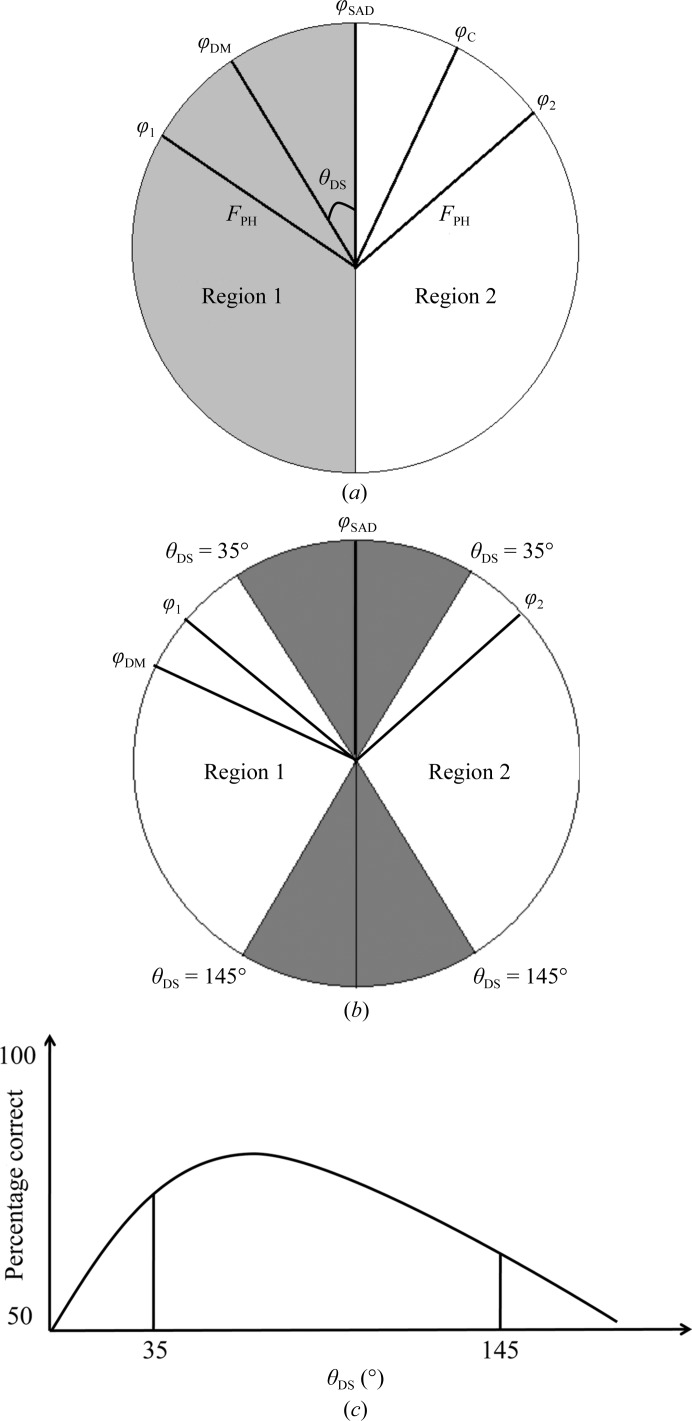
(*a*) Diagram of the various phases. The phase circle is divisible into two parts, with grey and white colours for regions 1 and 2, respectively. (*b*) Diagram of various phases and the θ_DS_ range. The circle is divided into grey and white. Angles θ_DS_ < 35° and θ_DS_ > 145° in the grey zone show the lower percentage correct in selecting phase ϕ_1_ or ϕ_2_ in region 1 or 2. (*c*) A schematic plot of the histograms of percentage correct as a function of the angle θ_DS_.

If the preliminary DM phase ϕ^NHL^_DM_ is located in region 1 or 2, the new initial ϕ_am_ phase is selected as ϕ_1_ or ϕ_2_, respectively (Fig. 4 ▶ *a*). The correct or incorrect selection is defined when ϕ^NHL^_DM_ and the model-calculated ϕ_am_ are in the same region or in different regions, respectively. The selected correct or incorrect phase ϕ_am_ (ϕ_1_ or ϕ_2_) is thus based on the phase ϕ^NHL^_DM_ because the model phase ϕ_C_ is fixed by the refined structures. Here, we define the percentage correct as the number ratio of the reflections with selected correct phases to the total reflections. The percentage correct and the angle θ_DS_ define the ‘confidence level’ and the ‘confidence interval’, respectively.

The protocol to determine the percentage correct for the simulation cases is applicable not only to the direct phase-selection method in the experimental group but also to the regular and non-constraint methods in the control group. However, ϕ_C_ is not available from the practical cases without known structures to distinguish the correct or incorrect phase ϕ_am_. The distribution statistics of the percentage correct from our five simulation cases enable us to instead use the novel ‘θ_DS_ list’ as the criterion to select the phases for the practical cases without structural models. The details of experimental procedures utilizing the θ_DS_ list in the direct phase-selection method *de novo* are described in the following sections.

#### 3.4.4. Direct phase-selection method based on θ_DS_ angles   

Our simulation results and statistics show that a high percentage correct occurs at an angle θ_DS_ in the range between 35 and 145° in regions 1 and 2 (Figs. 4 ▶ *c* and 5 ▶). A higher confidence level is hence obtained in the confidence interval between 35 and 145° in regions 1 and 2. A ‘θ_DS_ list’ from the smallest to the largest angles can be generated. Reflections from the θ_DS_ list with the angle θ_DS_ between 35 and 145° are selected, which have a relatively high probability of the correct selected phase ϕ_am_. The selected phase ϕ_am_ is either ϕ_1_ or ϕ_2_ depending on the preliminary DM phase ϕ^NHL^_DM_. The initial phases ϕ_SAD_ of all of the reflections in the range 35–145° are then replaced by the corresponding selected phases ϕ_am_ for optimized improvement.

The reflections with replaced phases (selected phases ϕ_am_) and the rest with unselected initial phases ϕ_SAD_ are subsequently combined into a new data set with optimum initial phases ϕ^S^_SAD_. In this step, FOM = 1.0 was used as the weighting scheme for the selected phase ϕ_am_ without Hendrickson–Lattman coefficients, whereas the initial FOM values were used for the rest of the unselected phases. In the DM process no phase recombination was carried out, only use of the FOM as the weighting scheme. The final DM phase ϕ^S^_DM_ was improved from ϕ^S^_SAD_ using *RESOLVE* or *DM* in parallel. After the above calculation, final DM phases ϕ^S^_DM_ from the direct phase-selection method can be obtained for the calculation of electron density and the evaluation of the percentage correct. Optimum initial phases ϕ^S^_SAD_ possessing a higher percentage correct have a better chance of improving the DM phases ϕ^S^_DM_ compared with ϕ^R^_DM_ and ϕ^N^_DM_ in the control group. This method is called the ‘direct phase-selection method’.

## 4. Results   

### 4.1. Determination of heavy-atom substructures   

For five test cases, the substructures in protein crystals were first solved with *SHELXC*/*D*/*E* based on the anomalous difference maps of each SAD data set. The numbers of heavy-atom sites and sulfur ‘super-atom’ sites were determined (Fig. 3 ▶). Five and three strong sulfur ‘super-atom’ sites with occupancies greater than 0.75 and 0.60 were located in the unit cells of lysozyme_S and insulin_S at resolutions of 1.82 and 2.52 Å, respectively. Two Zn positions with occupancies near 1 were determined in lectin_Zn. One Gd position with occupancy ∼1 was found in lysozyme_Gd. Eight Fe sites with occupancies greater than 0.8 were located in cytochrome *c*_3__Fe. All of the sites with occupancies found by *SHELXC*/*D*/*E* were input directly to *Phaser* in *CCP*4 to generate the initial SAD phases ϕ_SAD_. The overall initial 〈FOM_SAD_〉 (mean FOM) of the five test cases were determined as 0.489, 0.262, 0.553, 0.467 and 0.543 for cytochrome *c*_3__Fe, lectin_Zn, lysozyme_Gd, lysozyme_S and insulin_S, respectively.

### 4.2. Relationship between the percentage correct and the angle θ_DS_   

Based on the simulation results using the direct phase-selection method (§[Sec sec3.4]3.4) and the model phases ϕ_C_, the statistics clearly show that the percentage correct at angles θ_DS_ in the range between 35 and 145° is generally higher than that at other angles (Figs. 4 ▶ *c* and 5 ▶). In five simulation cases, the percentage correct could not be efficiently estimated in the θ range 0–10° for lectin_Zn, lysozyme_Gd, lysozyme_S and insulin_S because there are either no or only a few reflections in this small range.

### 4.3. The percentage correct *versus* the initial FOM using various methods   

In this section, we show how the relation between the initial FOM and the percentage correct varies according to the three methods. In five simulation cases, the data sets from the regular method, the non-constraint method and the direct phase-selection method show that the percentage correct varies with the range of the initial FOM (Fig. 6 ▶). After calculations with the regular method, the non-constraint method and the direct phase-selection method, some parameters, including the final DM phase and the model-calculated phase ϕ_C_, are used to evaluate the percentage correct for each case for comparison purposes. A correct or incorrect selection is defined as when the final DM phase (ϕ^R^_DM_, ϕ^N^_DM_ or ϕ^S^_DM_) and the model-calculated ϕ_C_ are in the same region or are in different regions, respectively. The percentage correct is defined as the number ratio of reflections with selected correct phases to the total reflections. A comparison of the same reflections in each FOM interval among the data sets from the regular method, the non-constraint method and the direct phase-selection method indicates that the percentage correct using the direct selection method is higher than those of the regular and non-constraint methods in all five cases. The percentage correct with the regular method is slightly higher than that with the non-constraint method in each case (Fig. 6 ▶).

### 4.4. Improvement of density-map quality   

In this section, we examine the differences among the regular map, the non-constraint map and the direct phase-selection map after final density modification with *RESOLVE*. A comparison of the regular maps, non-constraint maps and the direct phase-selection maps in all five test cases shows that the continuity and completeness of the electron-density maps using the direct phase-selection method are significantly improved and are superior to those of the regular map and the non-constraint map (Fig. 7 ▶). The statistical indicators, such as the map correlation coefficient and mean phase error, for the map quality after DM are evaluated in Table 2 ▶. On comparison, the new direct phase-selection method gives better map quality statistics than those for the regular and non-constraint methods for all test cases.

Similarly, with the above-mentioned protocol but using the *CCP*4 program *DM* with solvent flattening, the results from all test cases show that the new selection method gives better statistics than those for the regular and non-constraint methods (Table 3 ▶).

### 4.5. A comparison of model building with regular, non-constraint and direct selection maps   

In our experiment, automated model building after the final density modification with *RESOLVE* for the five test cases was performed with *ARP*/*wARP*. According to the results of model building shown in Table 2 ▶, the completeness of autobuilt residues with main chains and side chains in lectin_Zn, lysozyme_Gd and lysozyme_S with the direct phase-selection method is greater than those with the non-constraint and regular methods. The autobuilding results for insulin_S are comparable among the three methods. For a parallel comparison, improvements in model building were also observed with the *CCP*4 program *DM* combined with the direct phase-selection method (Table 3 ▶).

All of the proteins could be autobuilt using *ARP*/*wARP* except for cytochrome *c*_3__Fe (resolution 3.0 Å) because of the resolution limitation of 2.5 Å for *ARP*/*wARP* autobuilding. The structure of cytochrome *c*_3__Fe could, however, be built manually (73%) based on the improved density map at resolution 3.0 Å generated from the direct phase-selection method compared with the maps from the regular and non-constraint methods, which were not suitable for model building because of severe discontinuity.

### 4.6. Application to an unknown structure   

In this section, we applied our newly investigated selection method to a practical case with an unknown structure: histidine-containing phosphotransfer domain B (HptB). HptB comprises 116 amino-acid residues with a molecular mass of ∼13.2 kDa. HptB_Se was expressed in *E. coli* in selenomethionine medium for Se-SAD phasing and structure determination. Crystals of HptB_Se diffracted to 2.0 Å resolution and exhibited the symmetry of space group *I*4_1_22 (Table 1 ▶). Interpretations of the anomalous difference map with *SHELXC*/*D*/*E *revealed nine Se sites with occupancies in the range 0.4–1.0 (Fig. 3 ▶). The overall 〈FOM_SAD_〉 of the initial SAD phases was determined to be 0.428 using *Phaser* in *CCP*4.

The preliminary DM phases (ϕ^NHL^_DM_) were obtained from the initial SAD phases after the first run of *DM* in *CCP*4. The θ_DS_ list from the smallest to the largest was then generated; the phase ϕ_1_ or ϕ_2_ in each reflection at a corresponding angle θ_DS_ in a range between 35 and 145° was subsequently selected with the direct phase-selection method. Data sets with optimized ϕ^S^_SAD_ were generated and subsequently directed to *RESOLVE* to calculate the direct selection map with improved final DM phases ϕ^S^_DM_ (Fig. 7 ▶). In this step, no phase combination was carried out, thus no Hendrickson–Lattman coefficients were provided; all of the data were used for density modification without a resolution cutoff. For comparison, the regular and non-constraint maps were also obtained with the regular and non-constraint methods, respectively, using *RESOLVE*. As a result, similar to the five test cases, the continuity and completeness of the direct selection map were significantly improved (Fig. 7 ▶).

The initial protein structure was then autobuilt with *ARP*/*wARP* and the final model was refined and completed with *REFMAC*5 and *Coot*. The results of the automated model building of the HptB_Se structure with *ARP*/*wARP* are compared among the various methods, which show that the direct selection method produces a much higher completeness of residues built with side chains and main chains (Table 2 ▶). The newly determined and refined structure of HptB allowed us to calculate the model phases (ϕ^C^) to interpret and to compare the regular method, the non-constraint method and the direct selection method with the statistics of map-quality indicators using *RESOLVE*. According to the comparison, the quality of the electron-density map using our new direct phase-selection method is much improved, with superior statistics for indicators including the map correlation coefficient, the mean phase error and the completeness of built residues (Table 2 ▶ and Fig. 7 ▶). For a comparison, improvements were also obtained with the *CCP*4 program *DM* combined with the direct phase-selection method (Table 3 ▶).

A few more test examples, including chitinase with Zn atoms (Hsieh, Wu *et al.*, 2010 ▶), sulfite reductase with Fe (Hsieh, Liu *et al.*, 2010 ▶) and the unknown structure of haemerythrin with Fe (Phimonphan *et al.*, unpublished data), have also been applied and examined using our new phase-selection method. All of the results showed that the direct phase-selection method produced a similar improvement as in previously described cases, with enhanced electron densities and statistics of indicators (Supplementary Table S1).

## 5. Discussion   

### 5.1. Simulated phase selection based on the model phase ϕ_C_   

According to the direct phase-selection method, a portion of the ϕ_am_ (ϕ_1_ or ϕ_2_) phase set can be correctly selected with preliminary DM phases ϕ^NHL^_DM_. Our ultimate objective is to select the correct phase (ϕ_1_ or ϕ_2_) optimally. In our simulation experiment, we demonstrate that the correct phase (ϕ_1_ or ϕ_2_ can be effectively selected with the model phase ϕ_C_ for each reflection in all simulation cases. The correct selected phases ϕ_am_ are hence highly dependent on the phases ϕ_C_. The mean phase errors of the final DM phases and the map correlation coefficients of both the initial phases and the final DM phases were calculated for five simulation cases for evaluation (Table 2 ▶). A comparison of the results clearly shows that the simulation with known ϕ_C_ produces much improved map correlation coefficients and mean phase errors (by 0.05–0.3 and 10–35°, respectively) relative to the regular and non-constraint methods with *RESOLVE*. A similar improvement of map correlation coefficients and mean phase errors was observed using the direct selection method combining the *CCP*4 program *DM* with solvent flattening and standard parameters (Table 3 ▶). This simulation provides a basis for the use of the correctly selected initial phases to improve the map correlation coefficients and mean phase errors. However, ϕ_C_ is unavailable in practical cases without known structures for the selection of the correct phase ϕ_am_. The derivative direct phase-selection method using the angle θ_DS_, without the information of ϕ_C_, is hence investigated to improve the electron-density map for practical applications.

### 5.2. Comparison of the quality of density maps with various methods   

In all test cases, the electron-density map from our new direct phase-selection method is significantly better than those from conventional approaches in terms of map continuity and completeness (Fig. 7 ▶). The map correlation coefficients and mean phase errors are generally improved by 0.05–0.2 and 10–18°, respectively, using the direct phase-selection method with a single cycle utilizing the new selected phases ϕ_am_ (ϕ_1_ or ϕ_2_), with a higher confidence level to replace the corresponding initial SAD phases ϕ_SAD_ (Table 2 ▶). An iterative calculation with several cycles was also performed to examine any improvement; however, we found that the direct phase-selection method with two or three cycles did not show a notable improvement in map quality and indicators. All of the simulation results show that using selected phases ϕ_am_ (ϕ_1_ or ϕ_2_) based on the preliminary DM phase ϕ^NHL^_DM_, instead of ϕ_C_, could efficiently improve the map correlation coefficients and mean phase errors and generate density maps of a higher quality from the final DM phases ϕ^S^_DM_ compared with the regular methods with the Hendrickson–Lattman coefficients and the commonly used DM default procedures.

To further demonstrate the power of the direct phase-selection method, calculations using the regular method with combinations of phase choice by *OASIS* direct methods and density modification with *RESOLVE* and *DM* were carried out for comparison. In general, the regular method with *OASIS* initial phases and *DM* produced results better than those with *OASIS* initial phases and *RESOLVE*. However, our direct phase-selection method gave results that were superior overall to calculations using *OASIS* initial phases combined with both DM programs (Supplementary Table S2).

Moreover, from analysis of our direct phase-selection method, selecting one of the two phase choices from the SAD phase probability distribution seems to shift the starting phase more than performing phase combination. Thus, we performed solvent flipping, another over-shifting method, for comparison. The results showed that using solvent flipping in the regular method did not produce better results than the direct selection method in terms of the mean phase errors and residues built (Supplementary Table S3).

### 5.3. Comparison of the map quality with various aspects of the direct selection method   

**Figure 5:**
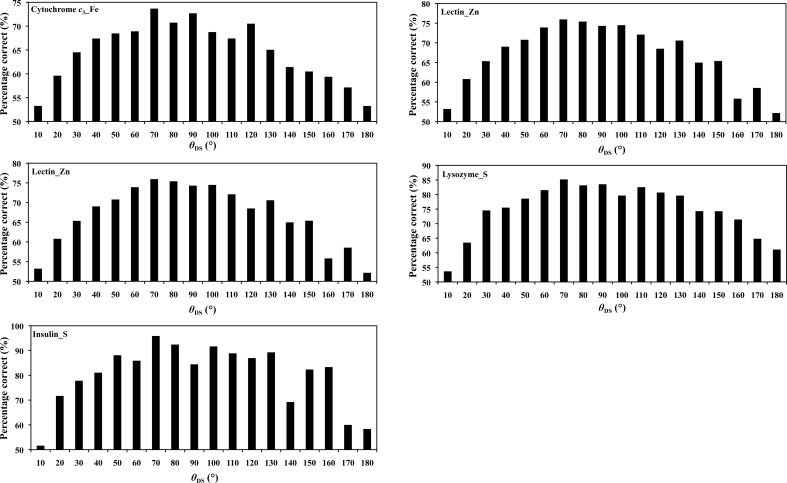
The highest percentage correct occurs at an angle θ_DS_ in the range between 35 and 145° for the selected phase ϕ_1_ or ϕ_2_ in region 1 or 2, respectively. The horizontal axis indicates the range of the angle θ_DS_ from 0.1 to 180°, whereas the vertical axis indicates the percentage correct.

To optimize the algorithm for direct phase selection, we performed a parallel comparison with various aspects related to this method, including FOM weighting, resolution, iterative cycles, initial SAD phases and ranges. For the FOM, we performed a parallel comparison of three different FOM weighting schemes in our direct phase-selection method: (i) FOM = 1.0 for the selected phases and the initial FOM from SAD for the unselected phases, (ii) FOM = 1.0 for all phases and (iii) the initial FOM from SAD for all corresponding phases. From the parallel comparison, the direct selection method with the weighting scheme (i), which is used in this study, is either comparable to or better than the other two weighting schemes (ii) and (iii) (Supplementary Table S4). We also examined other possible weighting schemes coupled with the selection criteria, *i.e.* the percentage correct. Fig. 5 ▶ shows that the percentage correct varies at different ranges of θ_DS_. We tested the weighting scheme corresponding to the percentage correct for selected reflections in the range 35–145°. The respective FOM values are calculated based on the ratio of percentage correct for reflections in different θ_DS_ ranges. For the unselected reflections, the FOMs are set to the initial FOM values from SAD phasing. The comparison shows that the weighting scheme (i) with FOM = 1 for the directly selected phases is either better than or comparable to the percentage correct-dependent FOM weighting scheme.

For the resolution, we examined various resolution ranges varying from the highest resolution of the data and found no notable differences in improvement. For the iterative cycles, a series of iterative cycles were performed to examine any improvement with the direct phase-selection method, which showed that two or three more cycles did not produce a significant further improvement of the map quality and indicators. For the SAD initial phases, we tested the initial SAD phases generated from *OASIS* (He *et al.*, 2007 ▶) and performed the same protocols of the direct phase-selection method. The results show that our direct selection method using initial SAD phases generated directly from *Phaser* was better than using initial phases from *OASIS*. The details of the ranges used are described in the following section.

### 5.4. Percentage correct as a function of the angle θ_DS_   

For determination of the optimized range in this work, we extensively analyzed all of the calculations in various ranges for all test cases (Supplementary Table S5). The statistical indicators, including the map correlation coefficients, the mean phase errors and the completeness of the built residues, were improved in all cases with reflections with selected phases ϕ_am_ at θ_DS_ angles in the range 35–145° (except for lectin_Zn, where the angles were in the range 40–140°) relative to the other angle ranges. Considering the comparable statistical indicators (mean phase error 54.28° *versus* 54.14°) and the completeness of the built residues (84.3% *versus* 84.9% for main chains and 80.8% *versus* 80.5% for side chains) using the θ_DS_ ranges 35–145° and 40–140°, respectively (Supplementary Table S5), we suggest selecting the reflection phases from angles in the range 35–145° for lectin_Zn as in the other cases for unanimity based on statistical analysis. The fraction of selected reflections is about 0.28–0.48 of the total reflections for θ_DS_ angles between 35 and 145° in all six cases.

θ_DS_ angles of <35° and >145° show a lower percentage correct for the selection of phase ϕ_1_ or ϕ_2_ in region 1 or 2. For cases with θ_DS_ < 35°, the angles between the preliminary DM phase ϕ_SAD_ and the initial SAD phase ϕ^NHL^_DM_ might be too small to resolve the ambiguity from the two initial phases (ϕ_1_ and ϕ_2_) in the grey zone with a low percentage correct (Figs. 4 ▶ *b* and 4 ▶ *c*). Similarly, for cases with θ_DS_ > 145° (the grey zone), the angles between ϕ^NHL^_DM_ and ϕ_SAD_ might be too large such that the two initial phases (ϕ_1_ and ϕ_2_) cannot be effectively distinguished for the correct or incorrect phases with a low percentage correct.

We found that a higher average intensity 〈*I*〉 commonly corresponds to an angle θ_DS_ in the range between 40° and 120° (Supplementary Table S6). Some individual strong reflections have been shown to improve the map quality after density modification (Uervirojnangkoorn *et al.*, 2013 ▶; Vekhter, 2005 ▶; Zhang & Main, 1990 ▶). The distribution of strong reflections might be one of the reasons why the higher percentage correct occurs at a θ_DS_ angle in the range 35–145° (Figs. 4 ▶ *c* and 5 ▶). The algorithm of our direct selection method based on θ_DS_ angle combined with weighting schemes is different from previous methods.

### 5.5. Percentage correct *versus* initial FOM with various methods   

**Figure 6:**
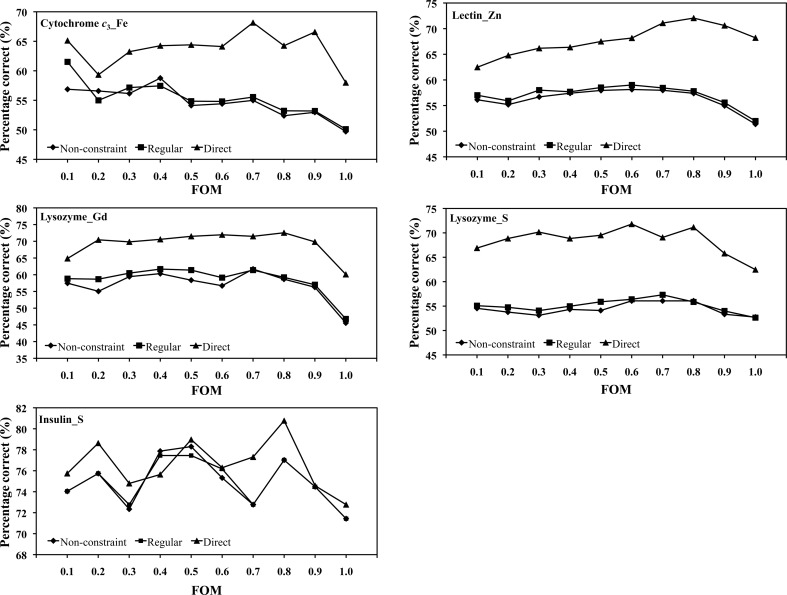
Percentage correct as a function of FOM for initial phases with the regular method, the non-constraint method and the direct phase-selection method. The horizontal axis indicates the initial FOM from the smallest to the largest values (0–1.0).

**Figure 7:**
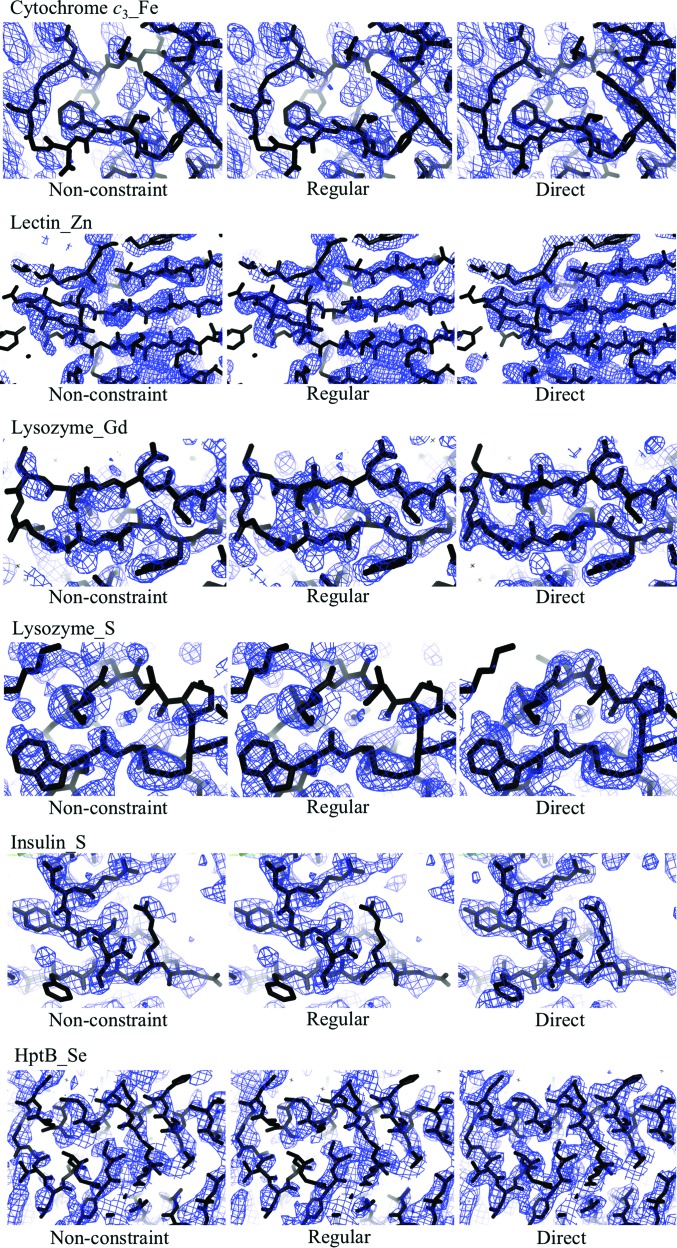
Electron-density maps of cytochrome *c*_3__Fe, lectin_Zn, lysozyme_Gd, lysozyme_S, insulin_S and HptB_Se (the unknown structure) after density modification with *RESOLVE* from various methods (the non-constraint map, the regular map and the direct selection map) are shown with the same contour level 1.0σ in blue. The corresponding structures are shown as black sticks.

A comparison of reflections in the same batch among different data sets for the regular method, the non-constraint method and the direct phase-selection method shows that the percentage correct with the direct phase-selection method is generally 5–10% higher than that with the regular and non-constraint methods in the five simulation cases (Fig. 6 ▶). Using the selected phases ϕ_am_ (ϕ_1_ or ϕ_2_) could thus efficiently improve the percentage correct compared with the regular method with the Hendrickson–Lattman coefficients and the commonly used DM procedure as described in §[Sec sec2]2. The percentage correct generally decreases for reflections with large initial FOM values (>0.8), which might result from the two close phases (ϕ_1_ and ϕ_2_), similar to cases with θ_DS_ < 35°. The percentage correct might also be affected by lack of closure, random errors and systematic errors (Borek *et al.*, 2003 ▶).

## 6. Conclusions   

The discussions above clearly show that the new procedure of phase improvement, *i.e.* the direct phase-selection method, combined with *RESOLVE* or the *CCP*4 program *DM*, can effectively improve the phase in comparison to the regular method with Hendrickson–Lattman coefficients using *RESOLVE* and *DM*. In the direct selection method, the SAD standard protocol was applied in the *RESOLVE* routine except for the Hendrickson–Lattman coefficients and fom_cut parameters. Similar improvements in SAD phases and density maps were obtained using *DM* with standard parameters except for histogram matching and Hendrickson–Lattman coefficients.

Ideally, according to our simulation study, a relatively high completeness for the selection of the correct phases ϕ_1_ or ϕ_2_ could be achievable, but only based on known ϕ_C_. A lack of known structures or model-calculated ϕ_C_ in the practical applications led us to investigate the novel ‘direct phase-selection method’, which utilizes the ‘θ_DS_ list’ to select phases ϕ_am_ from ϕ_1_ or ϕ_2_ of selected reflections (28–48%) with high percentage correct phases to replace the corresponding initial SAD phases ϕ_SAD_. A comparative analysis implies that the choice of a proper subset of reflections with the selected phase based on θ_DS_ might be more decisive than other aspects, such as the DM program and weighting scheme, in which FOM = 1.0 is used for the selected phases and the initial FOM of SAD is used for the unselected phases in this method.

Optimization of the initial phasing is considered to be a decisive factor in the success of the subsequent electron-density modification, model building and structure determination with the SAD method. Our new direct phase-selection method provides a powerful protocol with an essential additional selection step, combined with current DM software for simple solvent flattening, such as *RESOLVE* and *DM*, to resolve the initial phase ambiguities of a subset of reflections for further density modification. In contrast to most phase-improvement studies, which focus on density modification after the initial SAD phasing, our method focuses on the optimization of the initial phasing of a subset of reflections by imposing a binary phase choice, without using phase combination, to shift the phase probability distribution towards the better phase choice. With better initial SAD phases before carrying out the general DM procedure, the success rate of structure determination might be increased. The resulting final DM phases and electron-density maps were effectively improved by the direct phase-selection method compared with the regular method with Hendrickson–Lattman coefficients, yielding improved statistical indicators of map quality and completeness of model building. Based on our test results, with data of average or below average quality (high *R*_merge_ or medium–low resolution), the direct phase-selection method with an additional selection step for simple solvent flattening could still perform well with good electron density for model building. Optimization and increased completeness of the phase selection will be studied systematically in the near future.

## Supplementary Material

Supporting Information.. DOI: 10.1107/S1399004714013868/mh5112sup1.pdf

## Figures and Tables

**Table 1:** Statistics of X-ray data and structure refinement Values in parentheses are for the outermost shell.

Crystal	Cytochrome *c*_3_ (Fe-SAD)	Lectin (Zn-SAD)	Lysozyme (Gd-SAD)	Lysozyme (S-SAD)	Insulin (S-SAD)	HptB (Se-SAD)
Wavelength (Å)	1.73	1.282	1.712	1.55	1.77	0.97
Temperature (K)	110	110	110	110	110	110
Resolution range (Å)	30.0–3.00	30.0–1.90	50.0–2.00	30.0–1.82	30.0–2.52	30.0–2.00
Space group	*P*3_1_	*P*3_1_	*P*4_3_2_1_2	*P*4_3_2_1_2	*I*2_1_3	*I*4_1_22
Unit-cell parameters (Å)						
*a*	56.84	97.88	78.82	79.33	78.33	120.54
*b*	56.84	97.88	78.82	79.33	78.33	120.54
*c*	95.61	44.61	37.07	37.15	78.33	162.56
Unique reflections	6956	37476	8228	11111	2793	35118
Completeness (%)	100 (100)	99.2 (93.5)	99.8 (97.9)	99.7 (99.7)	99.1 (93.3)	91.6 (90.4)
〈*I*/σ(*I*)〉	22.6 (8.2)	28.2 (3.0)	40.3 (19.4)	79.6 (38.8)	89.1 (54.3)	21.01 (5.6)
Average multiplicity	11.6	8.9	17.2	37.5	20.7	13.4
*R*_merge_ † (%)	16.4	5.7	6.1	4.6	3.1	11.5
Refinement						
*R*_work_ ‡ (%)	18.8	18.9	16.0	17.4	17.6	20.8
*R*_free_ § (%)	28.1	23.0	24.2	23.0	22.4	25.9
R.m.s.d., bond lengths (Å)	0.018	0.036	0.021	0.024	0.023	0.026
R.m.s.d., bond angles (°)	1.90	2.60	1.72	1.87	1.96	2.04
No. of amino acids	109	159	129	129	51	116
No. of molecules in asymmetric unit	2	2	1	1	1	3
Average *B* factor (Å^2^)	21.6	38.9	18.9	17.9	17.8	21.0

† *R*_merge_ = 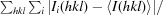 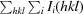, where *I_i_*(*hkl*) is the *i*th intensity measurement and 〈*I* *(hkl*)〉 is the weighted mean of all measurements of *I*(*hkl*). The reflection cutoff [*I*/σ(*I*) > 0] was applied in generating the statistics.

‡ *R*_work_ = 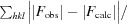 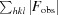, where *F*_obs_ and *F*_calc_ are the observed and calculate structure-factor amplitudes of reflection *hkl*.

§ *R*_free_ = 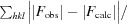 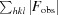 for 5% of the reserved reflections.

**Table 2:** Comparison of indicators of map quality among various methods with *RESOLVE*

		Initial phase map CC†		DM phase map CC†				
Data set	Method	M.C.	S.C.	M.C.	S.C.	Mean phase error‡ 〈Δϕ〉_DM_ (°)	Residues built with main chain§ (%)	Residues built with side chain§ (%)
Cytochrome *c*_3_ (Fe-SAD)	Non-constraint	0.544	0.441	0.560	0.438	63.19	—	—
	Regular	0.544	0.441	0.584	0.440	62.43	—	—
	Direct	0.575	0.490	0.624	0.547	52.07	—	—
	ϕ_C_ selection¶	0.677	0.596	0.737	0.656	28.07		
Lectin (Zn-SAD)	Non-constraint	0.617	0.339	0.589	0.332	72.02	40.8	8.8
	Regular	0.617	0.339	0.595	0.332	70.35	36.7	14.8
	Direct	0.717	0.470	0.836	0.613	54.28	84.3	80.8
	ϕ_C_ selection	0.840	0.592	0.901	0.692	32.72		
Lysozyme (Gd-SAD)	Non-constraint	0.657	0.445	0.610	0.384	55.12	0.0	0.0
	Regular	0.657	0.445	0.662	0.422	52.82	0.0	0.0
	Direct	0.731	0.533	0.808	0.655	41.01	90.7	90.7
	ϕ_C_ selection	0.779	0.607	0.807	0.673	28.16		
Lysozyme (S-SAD)	Non-constraint	0.618	0.425	0.437	0.344	62.31	10.4	8.5
	Regular	0.618	0.425	0.448	0.360	61.79	6.2	2.3
	Direct	0.698	0.492	0.774	0.620	44.67	93.8	93.8
	ϕ_C_ selection	0.775	0.568	0.821	0.671	29.26		
Insulin (S-SAD)	Non-constraint	0.610	0.497	0.750	0.653	34.34	90.2	90.2
	Regular	0.610	0.497	0.762	0.679	33.02	91.2	91.2
	Direct	0.672	0.573	0.773	0.684	30.12	92.8	92.8
	ϕ_C_ selection	0.735	0.631	0.773	0.670	23.70		
HptB (Se-SAD)	Non-constraint	0.590	0.406	0.611	0.428	55.54	58.6	12.6
	Regular	0.590	0.406	0.617	0.427	55.53	29.0	3.2
	Direct	0.641	0.538	0.781	0.622	38.14	87.6	85.1

† Map CC, map correlation coefficient; M.C., main chain; S.C., side chain.

‡ Mean phase error 〈Δϕ〉_DM_ = 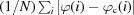, where ϕ(*i*) is the DM phase of the *i*th reflection and ϕ_c_(*i*) is the model phase. *N* donates the total number of reflections.

§ The completeness of autobuilt residues with side chains was calculated with *ARP*/*wARP*. All proteins can be auto-built except for cytochrome *c*_3__Fe, because the resolution limit of autobuilding in *ARP*/*wARP* is ∼2.5 Å.

¶ Phase ϕ_1_ or ϕ_2_ is selected based on the model phase ϕ_C_.

**Table 3:** Comparison of indicators of map quality among various methods with the *CCP*4 program *DM*

		Initial phase map CC†		DM phase map CC†				
Data set	Method	M.C.	S.C.	M.C.	S.C.	Mean phase error‡〈Δϕ〉_DM_ (°)	Residues built with main chain[Table-fn tfn10] (%)	Residues built with side chain[Table-fn tfn10] (%)
Cytochrome *c*_3_ (Fe-SAD)	Non-constraint	0.544	0.441	0.551	0.477	58.28	—	—
	Regular	0.544	0.441	0.554	0.475	58.48	—	—
	Direct	0.575	0.490	0.603	0.525	54.47	—	—
	ϕ_C_ selection[Table-fn tfn11]	0.677	0.596	0.719	0.636	31.01		
Lectin (Zn-SAD)	Non-constraint	0.617	0.339	0.720	0.481	64.61	78.3	73.8
	Regular	0.617	0.339	0.723	0.482	64.72	79.8	79.8
	Direct	0.717	0.470	0.772	0.536	57.08	84.0	80.8
	ϕ_C_ selection	0.840	0.592	0.883	0.668	28.00		
Lysozyme (Gd-SAD)	Non-constraint	0.657	0.445	0.715	0.514	49.93	91.4	91.4
	Regular	0.657	0.445	0.730	0.525	49.14	90.6	90.6
	Direct	0.731	0.533	0.774	0.596	44.51	95.4	95.4
	ϕ_C_ selection	0.779	0.607	0.794	0.636	30.03		
Lysozyme (S-SAD)	Non-constraint	0.618	0.425	0.681	0.499	52.29	96.1	96.1
	Regular	0.618	0.425	0.690	0.507	51.74	96.1	96.1
	Direct	0.698	0.492	0.747	0.561	47.03	96.8	96.8
	ϕ_C_ selection	0.775	0.568	0.809	0.627	30.67		
Insulin (S-SAD)	Non-constraint	0.610	0.497	0.730	0.622	37.16	90.4	90.0
	Regular	0.610	0.497	0.733	0.628	36.56	91.1	90.7
	Direct	0.672	0.573	0.741	0.632	36.06	91.4	90.8
	ϕ_C_ selection	0.735	0.631	0.764	0.658	24.78		
HptB (Se-SAD)	Non-constraint	0.590	0.406	0.795	0.579	44.04	85.2	84.1
	Regular	0.590	0.406	0.803	0.591	43.25	84.9	84.7
	Direct	0.641	0.538	0.821	0.613	40.11	88.5	85.6
	ϕ_C_ selection	0.745	0.589	0.769	0.625	26.30		

† Map CC, map correlation coefficient; M.C., main chain; S.C., side chain.

‡ Mean phase error 〈Δϕ〉_DM_ = 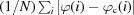, where ϕ(*i*) is the DM phase of the *i*th reflection and ϕ_c_(*i*) is the model phase. *N* donates the total number of reflections.

§ The completeness of autobuilt residues with side chains was calculated with *ARP*/*wARP*. All proteins can be auto-built except for cytochrome *c*_3__Fe, because the resolution limit of autobuilding in *ARP*/*wARP* is ∼2.5 Å.

¶ Phase ϕ_1_ or ϕ_2_ is selected based on the model phase ϕ_C_.

## Footnotes

1 Supporting information has been deposited in the IUCr electronic archive (Reference: MH5112).
