# Supplementary material for: Direct phase selection of initial phases from single-wavelength anomalous dispersion (SAD) for the improvement of electron density and ab initio structure determination
Source: Acta Crystallogr D Biol Crystallogr. 2014 Aug 29;70(Pt 9):2331–43. doi: 10.1107/S1399004714013868 (PMC4157445; doi:10.1107/S1399004714013868)
Supplement: Supplementary file 1 [file d-70-02331-sup1.pdf]

Supplementary Material

Manuscript title: Direct phase selection of initial phases from single-wavelength anomalous dispersion for the improvement of electron density and *ab initio* structure determination

Authors: Chung-De Chen, Yen-Chieh Huang, Hsin-Lin Chiang, Yin-Cheng Hsieh, Hong-Hsiang Guan, Phimonphan Chuankhayan, Chun-Jung Chen*

Supplementary List S1

SAD Data

Data collection: *.sca (SAD anomalous data)

$\Delta F^{\pm}$

SAD anomalous data includes the anomalous structure factor.

X_H

CCP4 SHELXC/D/E

The S and heavy-atom substructures (X_H) were determined from the anomalous SAD data ($F^{\pm}$).

Input: *.sca (SAD anomalous data)

Output: *.pdb (S or heavy-atom substructures positions and occupancy)

CCP4 Phaser

Initial SAD phases (ϕ_{SAD}) calculated and generated.

Input: *.mtz (SAD anomalous data)

*.pdb (S or heavy-atom substructures positions and occupancy)

Output: *.mtz (Data of initial SAD phase)

Control group

Regular map (Regular method)

In parallel, we used program *RESOLVE* and *CCP4* program *DM*, respectively, to improve the initial SAD phase to obtain the final DM phases (ϕ_{DM}^{R}) and the Regular map for the Regular method.

1a. *RESOLVE* program

Input: *.mtz (Data of initial SAD phase)

Output: *.mtz (Data of final DM phase)

Using the SAD standard protocol, including the Hendrickson-Lattman coefficients and fom_cut parameters.

1b. *CCP4* program *DM*

Input: *.mtz (Data of initial SAD phase)

Output: *.mtz (Data of final DM phase)

Solvent flattening alone or in combination with the histogram matching was used with the standard parameters, including the Hendrickson-Lattman coefficients with all reflections for the entire calculation.

After above calculations, an adapted data set for the Regular method was generated that included some important parameters from the mathematical operations, which include *hkl*, F_{hkl}, $_{SAD}$, initial FOM, ϕ_{DM}^{R} (final DM phase from the Regular method), θ, ϕ_{am}^{R} (ambiguity phase ϕ_1 or ϕ_2 determined by the final DM phase ϕ_{DM}^{R}), ϕ_1 and ϕ_2, θ_{DS}^{R} (the angle between the initial SAD phase ϕ_{SAD} and final DM phase ϕ_{DM}^{R}), and ϕ_C for the further evaluation of *percentage of correct*. Such an adapted data set is defined as "Regular data set".

Control group

Non-constraint map (Non-constraint method)

In parallel, we used program *RESOLVE* and *CCP4* program *DM*, respectively, to improve the initial SAD phase to obtain the final DM phases (ϕ_{DM}^{N}) and the Non-constraint map for the Non-constraint method.

2a. *RESOLVE* program

Input: *.mtz (Data of initial SAD phase)

Output: *.mtz (Data of final DM phase)

Using the SAD standard protocol, except the Hendrickson-Lattman coefficients and fom_cut parameters, which set the initial resolution for density modification to be at the point at which the FOM value is 0.

2b. *CCP4* program *DM*

Input: *.mtz (Data of initial SAD phase)

Output: *.mtz (Data of final DM phase)

Using the standard parameters and the phase extension in FOM steps. The histogram matching and the Hendrickson-Lattman coefficients were excluded.

After above calculations, an adapted data set for the Non-constraint method was generated that included some important parameters from the mathematical operations, which include *hkl*, F_{hkl}, ϕ_{SAD}, initial FOM, ϕ_{DM}^{N} (final DM phase from the Non-constraint method), θ, ϕ_{am}^{N} (ambiguity phase ϕ_1 or ϕ_2 determined by the final DM phase ϕ_{DM}^{N}), ϕ_1 and ϕ_2, θ_{DS}^{N} (the angle between the initial SAD phase ϕ_{SAD} and final DM phase ϕ_{DM}^{N}), and ϕ_C for the further evaluation of *percentage of correct*. Such an adapted data set is defined as “Non-constraint data set”.

Experimental group

Direct selection map (Direct selection method)

CCP4* program *DM

Input: *.mtz (Data of initial SAD phase)

Output: *.mtz (Data of preliminary DM phase)

Using the solvent flattening, histogram matching and all reflections for the entire calculation, involving no Hendrickson-Lattman coefficients (*NHL*).

The important angle parameters were then generated in the data sets for simulation examination, such as *hkl*, F_{hkl}, ϕ_{SAD}, initial FOM, ϕ_{DM}^{NHL} (preliminary DM phase), θ, ϕ_{am} (ambiguity phase ϕ_1 or ϕ_2 determined by the preliminary DM phase ϕ_{DM}^{NHL}), ϕ_1 and ϕ_2, θ_{DS} (the angle between the initial SAD phase ϕ_{SAD} and preliminary DM phase ϕ_{DM}^{NHL}) and ϕ_C for the further evaluation of *percentage of correct*. This data set is defined as "simulation data set".

Direct phase selection method

Input: *.dat (Simulation data set)

Output: *.mtz (Data of optimum initial phase)

In parallel, we used program *RESOLVE* and *CCP4* program *DM*, respectively, to improve the optimum initial SAD phase to obtain the final DM phases (ϕ_{DM}^{S}) and the Direct selection map for the Direct phase selection method.

3a. *RESOLVE* program

Input: *.mtz (Data of optimum initial SAD phase)

Output: *.mtz (Data of final DM phase)

Using the SAD standard protocol, except the Hendrickson-Lattman coefficients and fom_cut parameters, which set the initial resolution for density modification to be at the point at which the FOM value is 0.

3b. *CCP4* program *DM*

Input: *.mtz (Data of optimum initial SAD phase)

Output: *.mtz (Data of final DM phase)

Using the standard parameters and the phase extension in FOM steps. The histogram matching and the Hendrickson-Lattman coefficients were excluded.

After above calculations, an adapted data set for the Direct phase selection method was generated that included some important parameters from the mathematical operations, which include *hkl*, F_{hkl}, ϕ_{SAD}, initial FOM, ϕ_{DM}^{S} (final DM phase from the Direct phase selection method), θ, ϕ_{am}^{S} (ambiguity phase ϕ_1 or ϕ_2 determined by the final DM phase ϕ_{DM}^{S}), ϕ_1 and ϕ_2, θ_{DS}^{S} (the angle between the initial SAD phase ϕ_{SAD} and final DM phase ϕ_{DM}^{S}), and ϕ_C for the further evaluation of *percentage of correct*. Such an adapted data set is defined as “Direct selection data set”.

Supplementary Table S1. A comparison of map-quality indicators of test data sets among various methods with *RESOLVE* program

Data set	Method	Initial phase Map c. c.[a]		DM phase Map c. c.[a]		Mean Phase error[b] $\langle \Delta\phi \rangle_{DM}$ (°)	Residues built with main chain[c] (%)	Residues built with side chain[c] (%)
		M. C.	S. C.	M. C.	S. C.			
Chitinase (Zn-SAD) (30.0−2.3Å)	Non-constraint	0.492	0.478	0.600	0.586	55.78	71.8	71.1
	Regular	0.492	0.478	0.602	0.590	51.66	72.2	71.9
	Direct	0.540	0.515	0.663	0.616	48.31	73.1	72.3
	ϕ_c selection[d]	0.594	0.568	0.691	0.646	30.01	83.5	81.4
Dsr (Fe-SAD) (30.0−2.6Å)	Non-constraint	0.206	0.106	0.210	0.191	79.04	-	-
	Regular	0.206	0.106	0.302	0.207	74.36	-	-
	Direct	0.311	0.253	0.409	0.323	66.51	-	-
	ϕ_c selection	0.325	0.277	0.456	0.398	63.89	-	-
McHr (Fe-SAD) (30.0−2.0Å)	Non-constraint	0.660	0.353	0.485	0.182	81.20	0	0
	Regular	0.660	0.353	0.543	0.231	78.15	0	0
	Direct	0.735	0.459	0.758	0.497	53.83	96.2	95.4
	ϕ_c selection	0.756	0.552	0.804	0.618	33.85	96.2	95.4

[a] Map c. c.: map correlation coefficient, M. C.: the main chain, S. C.: the side chain.

[b] Mean phase error $\langle \Delta\phi \rangle_{DM} = \frac{1}{N}\sum_i \left|\phi(i) - \phi_c(i)\right|$, where $\phi(i)$ is the DM phase of ith reflection and $\phi_c(i)$ is the model phase. *N* donates the total number of reflection.

[c] Completeness of auto-built residues with side chain is calculated with program *ARP/wARP*. All proteins can be auto-built, except Dsr_Fe, because the resolution limit of auto-building is ~ 2.5 Å in *ARP/wARP*.

[d] Phase ϕ_1 or ϕ_2 is selected based on the model phase ϕ_c.

Supplementary Table S2. A comparison of map-quality indicators among various methods. (regular method with *OASIS* initial phase vs. direct selection method)

Data set	Method	Initial phase Map c. c.[a]		DM phase Map c. c.[a]		Mean Phase error[b] $\langle \Delta\phi \rangle_{DM}$ (°)	Residues built with main chain[c] (%)	Residues built with side chain[c] (%)
		M. C.	S. C.	M. C.	S. C.			
Cyt. c_3 (Fe-SAD)	Regular (*Oasis+Resolve*)	0.458	0.377	0.478	0.427	68.90	-	-
	Regular (*Oasis+CCP4 DM*)	0.458	0.377	0.588	0.518	54.57	-	-
	Direct (*Resolve*)	0.575	0.490	0.624	0.547	52.07	-	-
	Direct (*CCP4 DM*)	0.575	0.490	0.603	0.525	54.47	-	-
Lectin (Zn-SAD)	Regular (*Oasis+Resolve*)	0.506	0.229	0.106	0.066	80.37	0.0	0.0
	Regular (*Oasis+CCP4 DM*)	0.506	0.229	0.745	0.537	59.61	80.2	77.0
	Direct (*Resolve*)	0.717	0.470	0.836	0.613	54.28	84.3	80.8
	Direct (*CCP4 DM*)	0.717	0.470	0.772	0.536	57.08	84.0	80.8
Lysozyme (Gd-SAD)	Regular (*Oasis+Resolve*)	0.484	0.235	0.187	0.040	85.89	0.0	0.0
	Regular (*Oasis+CCP4 DM*)	0.484	0.235	0.505	0.349	73.68	15.5	0.0
	Direct (*Resolve*)	0.731	0.533	0.808	0.655	41.01	90.7	90.7
	Direct (*CCP4 DM*)	0.731	0.533	0.774	0.596	44.51	95.4	95.4
Lysozyme (S-SAD)	Regular (*Oasis+Resolve*)	0.412	0.279	0.216	0.222	78.98	0.0	0.0
	Regular (*Oasis+CCP4 DM*)	0.412	0.279	0.555	0.386	69.78	20.1	0.0
	Direct (*Resolve*)	0.698	0.492	0.774	0.620	44.67	93.8	93.8
	Direct (*CCP4 DM*)	0.698	0.492	0.747	0.561	47.03	96.8	96.8
Insulin (S-SAD)	Regular (*Oasis+Resolve*)	0.018	0.172	0.144	0.200	77.12	0.0	0.0
	Regular (*Oasis+CCP4 DM*)	0.018	0.172	0.345	0.287	62.11	0.0	0.0
	Direct (*Resolve*)	0.672	0.573	0.773	0.684	30.12	92.8	92.8
	Direct (*CCP4 DM*)	0.672	0.573	0.741	0.632	36.06	91.4	90.8
HptB (Se_SAD)	Regular (*Oasis+Resolve*)	0.335	0.284	0.428	0.354	80.34	0.0	0.0
	Regular (*Oasis+CCP4 DM*)	0.335	0.284	0.785	0.562	49.77	85.9	31.6
	Direct (*Resolve*)	0.641	0.538	0.781	0.622	38.14	87.6	85.1
	Direct (*CCP4 DM*)	0.641	0.538	0.821	0.613	40.11	88.5	85.6

[a] Map c. c.: map correlation coefficient, M. C.: the main chain, S. C.: the side chain.

[b] Mean phase error $\langle \Delta\phi \rangle_{DM} = \frac{1}{N}\sum_i \left|\phi(i) - \phi_c(i)\right|$, where $\phi(i)$ is the DM phase of i th reflection and $\phi_c(i)$ is the model phase. N donates the total number of reflection.

[c] Completeness of auto-built residues with side chain is calculated with program *ARP/wARP*. All proteins can be auto-built, except cytochrome c_3_Fe, because the resolution limit of auto-building is ~ 2.5 Å in *ARP/wARP*.

[d] *CCP4 DM* : *CCP4* program *DM* Solvent flattening.

Supplementary Table S3. A comparison of map-quality indicators among various methods. (Regular method with solvent flattening, solvent flipping vs. Direct selection method)

Data set	Method	Initial phase Map c. c.[a]		DM phase Map c. c.[a]		Mean Phase error[b] $\langle\Delta\phi\rangle_{DM}$ (°)	Residues built with main chain[c] (%)	Residues built with side chain[c] (%)
		M. C.	S. C.	M. C.	S. C.			
Cyt. c_3 (Fe-SAD)	Regular (*CCP4 DM*)[d]	0.544	0.441	0.554	0.475	58.48	6.1	2.7
	Regular (*Resolve*)	0.544	0.441	0.584	0.440	62.43	-	-
	Regular (Solvent flipping)	0.544	0.441	0.629	0.527	58.19	-	-
	Direct (*Resolve*)	0.575	0.490	0.624	0.547	52.07	-	-
	Direct (*CCP4 DM*)	0.575	0.490	0.603	0.525	54.47	8.1	4.1
Lectin (Zn-SAD)	Regular (*CCP4 DM*)[d]	0.617	0.339	0.723	0.482	64.72	79.8	79.8
	Regular (*Resolve*)	0.617	0.339	0.595	0.332	70.35	36.7	14.8
	Regular (Solvent flipping)	0.617	0.339	0.811	0.554	63.69	75.2	75.2
	Direct (*Resolve*)	0.717	0.470	0.836	0.613	54.28	84.3	80.8
	Direct (*CCP4 DM*)	0.717	0.470	0.772	0.536	57.08	84.0	80.8
Lysozyme (Gd-SAD)	Regular (*CCP4 DM*)[d]	0.657	0.445	0.730	0.525	49.14	90.6	90.6
	Regular (*Resolve*)	0.657	0.445	0.662	0.422	52.82	0.0	0.0
	Regular (Solvent flipping)	0.657	0.445	0.765	0.596	54.75	50.4	50.4
	Direct (*Resolve*)	0.731	0.533	0.808	0.655	41.01	90.7	90.7
	Direct (*CCP4 DM*)	0.731	0.533	0.774	0.596	44.51	95.4	95.4
Lysozyme (S-SAD)	Regular (*CCP4 DM*)[d]	0.618	0.425	0.690	0.507	51.74	96.1	96.1
	Regular (*Resolve*)	0.618	0.425	0.448	0.360	61.79	6.2	2.3
	Regular (Solvent flipping)	0.618	0.425	0.715	0.552	57.58	41.2	40.1
	Direct (*Resolve*)	0.698	0.492	0.774	0.620	44.67	93.8	93.8
	Direct (*CCP4 DM*)	0.698	0.492	0.747	0.561	47.03	96.8	96.8
Insulin (S-SAD)	Regular (*CCP4 DM*)[d]	0.610	0.497	0.733	0.628	36.56	91.1	90.7
	Regular (*Resolve*)	0.610	0.497	0.762	0.679	33.02	91.2	91.2
	Regular (Solvent flipping)	0.610	0.497	0.752	0.658	32.18	92.2	92.2
	Direct (*Resolve*)	0.672	0.573	0.773	0.684	30.12	92.8	92.8
	Direct (*CCP4 DM*)	0.672	0.573	0.741	0.632	36.06	91.4	90.8
HptB (Se_SAD)	Regular (*CCP4 DM*)[d]	0.590	0.406	0.803	0.591	43.25	84.9	84.7
	Regular (*Resolve*)	0.590	0.406	0.617	0.427	55.53	29.0	3.2
	Regular (Solvent flipping)	0.590	0.406	0.768	0.614	46.24	55.4	54.4
	Direct (*Resolve*)	0.641	0.538	0.781	0.622	38.14	87.6	85.1
	Direct (*CCP4 DM*)	0.641	0.538	0.821	0.613	40.11	88.5	85.6

[a] Map c. c.: map correlation coefficient, M. C.: the main chain, S. C.: the side chain.

[b] Mean phase error $\langle\Delta\phi\rangle_{DM} = \frac{1}{N}\sum_i \left|\phi(i) - \phi_c(i)\right|$, where $\phi(i)$ is the DM phase of i th reflection and $\phi_c(i)$ is the model phase. N donates the total number of reflection.

[c] Completeness of auto-built residues with side chain is calculated with program *ARP/wARP*. All proteins can be auto-built, except cytochrome c_3_Fe, because the resolution limit of auto-building is ~ 2.5 Å in *ARP/wARP*.

[d] *CCP4 DM* : *CCP4* program *DM* Solvent flattening.

Supplementary Table S4. A comparison of map-quality indicators among various FOM weight schemes with direct selection method.

Data set	Method	Initial phase Map c. c.[a]		DM phase Map c. c.[a]		Mean Phase error[b] $\langle\Delta\phi\rangle_{DM}$ (°)	Residues built with main chain[c] (%)	Residues built with side chain[c] (%)
		M. C.	S. C.	M. C.	S. C.			
Cyt. c_3 (Fe-SAD)	Direct	0.575	0.490	0.624	0.547	52.07	-	-
	Direct (FOM=1)	0.561	0.474	0.601	0.523	54.78	-	-
	Direct (FOM=original)	0.587	0.493	0.636	0.550	51.14	-	-
Lectin (Zn-SAD)	Direct	0.717	0.470	0.836	0.613	54.28	84.3	80.8
	Direct (FOM=1)	0.717	0.466	0.812	0.588	58.61	81.2	79.9
	Direct (FOM=original)	0.692	0.490	0.838	0.621	52.58	84.8	82.4
Lysozyme (Gd-SAD)	Direct	0.731	0.533	0.808	0.655	41.01	90.7	90.7
	Direct (FOM=1)	0.702	0.504	0.733	0.556	56.06	47.2	46.5
	Direct (FOM=original)	0.725	0.521	0.787	0.631	49.86	91.5	91.5
Lysozyme (S-SAD)	Direct	0.698	0.492	0.774	0.620	44.67	93.8	93.8
	Direct (FOM=1)	0.666	0.472	0.676	0.525	58.39	30.7	24.8
	Direct (FOM=original)	0.688	0.481	0.676	0.521	56.99	23.2	15.5
Insulin (S-SAD)	Direct	0.672	0.573	0.773	0.684	30.12	92.8	92.8
	Direct (FOM=1)	0.555	0.475	0.682	0.574	47.37	92.2	92.2
	Direct (FOM=original)	0.611	0.497	0.750	0.653	39.10	90.1	90.1
HptB (Se_SAD)	Direct	0.641	0.538	0.781	0.622	38.14	87.6	85.1
	Direct (FOM=1)	0.463	0.431	0.761	0.592	45.54	88.5	86.2
	Direct (FOM=original)	0.462	0.428	0.782	0.625	40.17	89.1	87.9

[a] Map c. c.: map correlation coefficient, M. C.: the main chain, S. C.: the side chain.

[b] Mean phase error $\langle\Delta\phi\rangle_{DM} = \frac{1}{N}\sum_i |\phi(i) - \phi_c(i)|$, where $\phi(i)$ is the DM phase of i th reflection and $\phi_c(i)$ is the model phase. N donates the total number of reflection.

[c] Completeness of auto-built residues with side chain is calculated with program *ARP/wARP*. All proteins can be auto-built, except cytochrome c_3_Fe, because the resolution limit of auto-building is ~ 2.5 Å in *ARP/wARP*.

Supplementary Table S5. A comparison of map indicators with various θ_{DS} range

Data set	θ_{DS} range	Initial phase Map c. c.[a]		DM phase Map c. c.[a]		Mean Phase error[b] $\langle\Delta\phi\rangle_{DM}$ (°)	Residues built with main chain[c] (%)	Residues built with side chain[c] (%)
		M. C.	S. C.	M. C.	S. C.			
Cyt. c_3 (Fe-SAD)	35°−105°	0.570	0.481	0.625	0.557	52.12	-	-
	30°−100°	0.567	0.482	0.616	0.543	52.81	-	-
	25°−115°	0.568	0.488	0.622	0.547	52.28	-	-
	20°−110°	0.565	0.487	0.621	0.546	52.53	-	-
	45°−95°	0.570	0.473	0.609	0.534	53.94	-	-
	40°−90°	0.567	0.475	0.613	0.534	53.95	-	-
	35°−130°	0.573	0.487	0.619	0.544	52.39	-	-
	35°−140°	0.575	0.488	0.622	0.545	52.21	-	-
	35°−145°	0.575	0.490	0.624	0.547	52.07	-	-
	40°−140°	0.573	0.487	0.622	0.543	52.48	-	-
Lectin (Zn-SAD)	35°−105°	0.700	0.447	0.838	0.612	55.27	84.2	84.2
	30°−100°	0.695	0.449	0.835	0.609	55.74	83.6	83.6
	25°−115°	0.701	0.463	0.836	0.613	55.33	81.1	78.6
	20°−110°	0.702	0.453	0.829	0.604	56.03	83.0	78.9
	45°−95°	0.688	0.427	0.838	0.612	55.75	84.2	84.2
	40°−90°	0.685	0.429	0.838	0.611	56.10	82.7	78.3
	35°−130°	0.714	0.465	0.836	0.615	54.54	82.3	79.2
	35°−140°	0.717	0.469	0.839	0.615	54.31	81.9	80.1
	35°−145°	0.717	0.470	0.836	0.613	54.28	84.3	80.8
	40°−140°	0.714	0.467	0.841	0.619	54.14	84.9	80.5
Lysozyme (Gd-SAD)	35°−105°	0.722	0.520	0.798	0.638	42.44	90.0	90.0
	30°−100°	0.719	0.519	0.799	0.639	42.47	90.2	90.2
	25°−115°	0.726	0.527	0.799	0.649	42.06	86.0	86.0
	20°−110°	0.731	0.526	0.802	0.649	42.24	90.6	90.6
	45°−95°	0.708	0.506	0.788	0.611	43.94	90.0	90.0
	40°−90°	0.706	0.500	0.787	0.622	43.24	90.7	90.7
	35°−130°	0.728	0.529	0.806	0.653	41.45	90.3	90.3
	35°−140°	0.729	0.532	0.805	0.654	41.22	90.7	90.7
	35°−145°	0.731	0.533	0.808	0.655	41.01	90.7	90.7
	40°−140°	0.726	0.526	0.805	0.650	41.29	90.5	90.5

Data set	θ_{DS} range	Initial phase Map c. c.[a]		DM phase Map c. c.[a]		Mean Phase error[b] $\langle \Delta\phi \rangle_{DM}$ (°)	Residues built with main chain[c] (%)	Residues built with side chain[c] (%)
		M. C.	S. C.	M. C.	S. C.			
Lysozyme (S-SAD)	35°−105°	0.691	0.483	0.771	0.619	45.21	92.6	92.6
	30°−100°	0.694	0.486	0.767	0.605	45.83	92.1	92.1
	25°−115°	0.699	0.492	0.769	0.618	45.24	93.0	93.0
	20°−110°	0.697	0.488	0.767	0.618	45.57	92.1	92.1
	45°−95°	0.676	0.476	0.726	0.561	48.25	93.1	93.1
	40°−90°	0.679	0.476	0.725	0.579	48.09	82.2	82.2
	35°−130°	0.696	0.490	0.725	0.581	44.75	91.0	91.0
	35°−140°	0.698	0.493	0.734	0.612	44.77	92.1	92.1
	35°−145°	0.698	0.492	0.774	0.620	44.67	93.8	93.8
	40°−140°	0.693	0.493	0.766	0.605	44.94	90.2	90.2
Insulin (S-SAD)	35°−105°	0.611	0.497	0.750	0.653	34.34	90.2	90.2
	30°−100°	0.613	0.498	0.751	0.654	34.31	90.3	90.3
	25°−115°	0.605	0.495	0.747	0.650	34.35	90.0	90.0
	20°−110°	0.609	0.496	0.749	0.651	35.30	90.2	90.2
	45°−95°	0.610	0.497	0.750	0.653	35.31	90.2	90.2
	40°−90°	0.614	0.498	0.752	0.655	33.38	90.5	90.5
	35°−130°	0.613	0.497	0.751	0.654	34.39	90.3	90.3
	35°−140°	0.614	0.498	0.752	0.655	34.26	90.5	90.5
	35°−145°	0.672	0.573	0.773	0.684	30.12	92.8	92.8
	40°−140°	0.615	0.498	0.761	0.662	33.78	91.2	91.2
HptB (Se_SAD)	35°−105°	0.653	0.483	0.777	0.617	38.51	91.4	87.9
	30°−100°	0.652	0.482	0.776	0.616	38.72	83.9	83.0
	25°−115°	0.663	0.490	0.779	0.619	38.45	90.8	86.8
	20°−110°	0.662	0.489	0.778	0.618	38.71	87.4	84.2
	45°−95°	0.641	0.468	0.774	0.615	38.88	88.8	86.8
	40°−90°	0.639	0.467	0.774	0.612	39.08	86.5	86.5
	35°−130°	0.661	0.494	0.778	0.620	38.19	86.2	85.0
	35°−140°	0.663	0.495	0.779	0.620	38.22	86.3	84.1
	35°−145°	0.641	0.538	0.781	0.622	38.14	87.6	85.1
	40°−140°	0.640	0.499	0.779	0.621	38.27	86.1	84.8

Supplementary Table S6. Distributions of the average intensities in various θ_{DS} ranges

Data sets		θ_{DS} range (°)								
		20	40	60	80	100	120	140	160	180
Cyt. c_3 (Fe-SAD)	$\langle I \rangle$ (%)[a]	80.4	97.3	100.0	93.2	88.4	84.1	83.7	81.9	77.3
Lectin (Zn-SAD)	$\langle I \rangle$ (%)	97.8	96.1	98.0	97.2	99.3	100.0	92.3	90.4	88.2
Lysozyme (Gd-SAD)	$\langle I \rangle$ (%)	86.8	92.4	79.3	81.1	100.0	91.3	88.6	90.3	89.0
Lysozyme (S-SAD)	$\langle I \rangle$ (%)	98.7	96.4	97.3	100.0	90.1	87.2	85.3	82.3	87.5
Insulin (S-SAD)	$\langle I \rangle$ (%)	80.9	82.7	71.8	84.5	100.0	80.9	99.2	69.0	62.7
HptB (Se-SAD)	$\langle I \rangle$ (%)	67.2	89.9	100.0	95.7	93.1	80.3	73.9	62.3	65.8

[a] Average intensity $\langle I \rangle = \frac{1}{N}\sum_i I(i)$, where $I(i)$ is the intensity of i th reflection on each θ_{DS} range. N is the number of reflections for each θ_{DS} range. The percentage of $\langle I \rangle$ is calculated as the 100 times the ratio of the average intensity in each θ_{DS} range to the highest average intensity.
